# Notices for July

**Published:** 1868-07

**Authors:** 


### NOTICES FOR JULY.

Broughton & Wyman, 13 Bible House, New York, have published a 40 ct. book entitled "Social Hints for Young Christians" in three Sermons, by Howard Crosby, Pastor of the Fourth Av. Presbyterian Church, New York. This is just such a book as those need who have recently joined the church; it lays down clear, scriptural principles of action in their intercourse with those around them ; in their associations, in their amusements and in their efforts to engage in personal endeavors to promote and extend the cause and best interests of religion ; it is a timely and valuable present to any young christian.

At the same place may be found all the publications of the American Tract Society, as also at 28 Cornhill, Boston, one of which is "The Curse," or the position in the world's history occupied by the race of man ; by Rev. Increase N. Tarbox. The great practical point is the inculcation of our duty at this time toward the "blacks."

"God's Word Written," 358 pp. 12 mo., published by the same Society, ($1.25) explaining and enforcing the inspiration of the Holy Scriptures ; by the Rev. Edward Garbett, M. A., Incumbent of Christ Church, Surbiton, Boyle Lecturer for '61, '62, '63, and select preacher to the University of Oxford. It will strengthen the faith of any christian who will read this book and nerve him up to a higher standard of piety and to a more direct and personal effort to "work while the day lasts," "for Him who died for them and washed them in his blood."

"The Redeemer," being a sketch of the history of redemption ; by Edmund De Presenseé ; translated from the second edition by Rev. J. H. Myers, D.D., 412 pp. 12mo., by the same Society. The book forms a sketch of the history of redemption as exhibited in the life of Jesus Christ ; it treats of the fall, the promise of Christ's coming, the preparation for it, his nature, his plan, his holiness, as a teacher, as a prophet, as a sacrifice, as a king. Those who delight to feed on the wonders of redeeming love will find in this delightful volume a feast of fat things, of wine upon the lees, well refined.

"Old Virginia!" the grand old state which erst was the mother of Presidents, with proper cultivation could be made the granary of half a continent ; its eastern shore would supply a nation with the best oysters in the world, while the farms, forest, mines and oil wells of the western portion, now called "West Virginia," are more valuable than a mine of gold. J B. Lippincott & Co. of Philadelphia have published a book written by J. R. Dodge of the United States Department of

Agriculture, gives a glimpse of its scenery, its population, with an exhibit of its industrial statistics ; it gives just such information as any enterprizing man of capital, whether in head, hands or purse would want to know, who contemplated the purchase of cheap but extremely valuable lands in that great State, with a view to develope the resources of the soil. No mere speculator is wanted there, but men of intelligence, of individual industry and indomitable perseverance.

The American Tract Society, 150 Nassau St. New York, have published in a 12mo of 150 pages, "The Life and times of Martin Luther," by W. Carlos Martyn, author of "the Life and Times of John Milton," intended to give an accurate view of Luther's character, and a clear idea of the grounds of the Reformation ; while literally and strictly true in all its details it is presented with the interest of an absorbing moral.

"Thoughts for Christians," by Rev. John Todd, D.D., in 32 chapters, each treating of a different subject of such practica character as occurs in what might be called the domestic history of any church, parish, congregation, or society ; it is a very instructive and suggestive volume, of 260 pages 12mo. Besides the above, the same Society has published

"Companion to the Bible," part 1, being evidences of revealed religion ; by Rev. E. P. Barrows, D. D., Professor of Biblical Theology.

"In the World, not of the World," being thoughts on Christian casuistry ; by William Adams, D.D., Madison Square church. "Love is the best casuist."

"Sketch of the religious character of Alexander, Emperor of Russia ;" by Rev. H. L. Empaytaz, Pastor of the Church of the Pelisserie, in Geneva, Switzerland ; translated from the French for the Christian Commission. The reverend author is a Swiss clergyman of high standing, remarkable for his piety and general excellence of character. The world at large only knows the Emperor Alexander as the magnificent autocrat of the Russian Empire, towering above his compeers in physical and mental proportions, as the wise statesman and the brave soldier. But Alexander stands out in a more striking light as an humble sincere and christian man, whose traits must be the same whether he be the king on his throne or the pauper of the poor house, whether he be a victorious general or a private soldier, a most impressive illustration of this levelling truth will be found in this very suggestive twenty cent volume, and if the reader will turn to the Dec. number for 1863, he will find similar proofs in the last sickness of Nicholas the 1st that kings lean trustingly on the Savior's arm when they pass through the valley and the shadow of death, and who does not know that his son and successor in the person of the present Autocrat, the Czar Alexander, has given an example of exalted heroism on the side of humanity in liberating many millions from a worse slavery than even our country witnessed by a single stroke of his pen, when such was the opposition on the part of the nobles and the high estates of the vast empire that all christian nations feared for him the loss of his throne; but fixing his eye on eternal right and equal justice he defied all opposition, risked his empire and his life and came off gloriously victorious, and long may he live, the august representative of a true humanity.

"The Bible True and Infidelity Wicked;" by Wm. J. Plummer, D.D.; a 15 cent work of 96 pages, written by one of the best of men and one of the ablest divines. We commend it to every reader.

"The Mind of Jesus;" by the author of "Morning and Night Watches," "The Words of Jesus," &c.

"The True Boy, or obstacles well met and ultimate triumph;" 15 cts. A right good present from a father who wants to bring up his little son to be of some account in the world.

"Little Robbie;" 13 pages; by Nellie Graham; eleven chapters, illustrating love, naughtiness, obedience, mischief, pleasure, &c. &c. "Words of Jesus," and "The United States Second Reading Book for the Freedmen."

Another admirable volume of 150 pages, 50 cts., is one by Rev. John Flavel, entitled "Jesus Christ's alluring love," being reasons for "coming to Jesus" the great physician of souls, the desire of all nations, and redeemer of the world.

" Colorado," or the Rocky Mountain Gem as it is in 1868; a gazeteer and handbook, describing its mineral and agricultural resources, with a lithographed map, by N. E. Farrell; published by the Western News Co., Chicago, Ill. To excursionists, and to all who think of moving westward, this 30 cent book will be found to contain a large amount of information of the kind required, as to its quartz, mining, coal, agriculture, homestead and preemption laws, who should go, summer trips, watering places, &c. &c.

The book of the season, to be read in all summer resorts, by-ways, highways and elsewhere, is the " Chimney Corner," by Mrs. Stowe, 311 pp. 12 mo., $1.75cts; issued by Ticknor & Fields, Boston. Perhaps the best "Notice" which could be given would be the table of contents. What will you do with woman? her sphere; family reconstruction; is woman a worker; the transition; good health; entertaining company; amusement; dress; fashion; sources of beauty in dress, &c. &c. A woman who could write a book (Uncle Tom's Cabin,) which the public would call for to the extent of three hundred and fourteen thousand copies up to May 1st, 1868, may well be supposed to be able to make the Chimney Corner the attraction of the hour.

" ALCOHOL, its nature and effects," in Ten Lectures, by Dr. Charles A. Story of Chicago, 392 pp. in large clear type giving a history of alcohol; where it comes from; its effects on the human body, on the mind, on society, &c. This is a book which every friend of temperance should read and study and master, it is an incontrovertible demonstration of the great temperance principles of the times.

" OUR PARISH," by Mrs. Emily Pearson. " This is another valuable addition to our steadily increasing library of standard temperance works for family reading. The manifold evils resulting from the " still " to the dealer's family, as well as to the families of his customers, are truthfully presented. The characters introduced, such as are found in almost every good-sized village, are well portrayed. The description given of the " sewing society," and the part it played in unsettling the minister, is very racy, and, as thousands can testify, truthful. We can most unhesitatingly commend it, and bespeak for it a wide circulation. Price, 75 cents."

"THE OLD BROWN PITCHER," by Miss Mary Dwinell Chellis, author of "Deacon Sims' Prayers" etc. This is a true story, replete with intrest, and adapted to Sunday-School and family reading. In it we have graphically depicted the sad ravages that are caused by the use of intoxicating beverages; also the blessings of Temperance, and what may be accomplished by one earnest soul for that reform. It ought to find readers in every household. $1.25. All the above books with many other equally useful publications are sold by A. C. Stearnes, publishing agent 127 William Street, New York, of the National Temperance Society.

AN ESSAY ON MAN. By Alexander Pope. With fifteen original illustrations, and Notes by S. R. Wells. One vol., 12mo, fancy cloth, beveled boards; gilt $1; paper, 50 cents. A succinct biography of the poet, and his highly esteemed "Universal Prayer," are published with the "Essay," making, together, a very desirable volume for the library or the center-table.

"HIGHER WATER." Longfellow's "Hiawatha" is known to fame. Within three days after its publication in the East there was read to a few friends on the bank of the Ohio one of the most humorous imitation that could possibly be conceived, being "The Song of Higher-Water" by James W. Ward, "Mus in gurgite flumenis," now first published by Robert H. Johnson & Co., New York, and Robert Clark & Co., Cincinnati, O. Whoever has read Hiawatha, will find an amount of mirth in "Higher-Water" which will impart more healthful influences to mind and body than any medica-mentum on the shelf of the Apothecary.

Apropos of Children's Teeth, tract 333 in this number, the "Dental Register of Cincinnati, Ohio," has an article on that subject by W. E. Magil, which is commended to the attention of every intelligent parent. Twenty-five cents could not be better expended than in the purchase of a copy instructing how to prevent poor teeth, and how best to remedy the misfortune, one idea in tract 333 will surprise many readers, that sweets do not directly injure the teeth ; yet it is true ; let the reader ask himself what positive proof have I in my own knowledge and observation that sweets injure the teeth more than anything else ; to attribute bad teeth to a wrong cause, leads to wrong practical results ; it is foul stomachs and unclean mouths which destroy the teeth. Robust health, outdoor exercise and coarse breads are the best tooth preservers in the world.

The Aeronautical Society of Great Britain, Duke of Argyle, Pres., Duke of Sutherland and Lord Grosvenor, Vice Prest's. has for its objects the reception and consideration of suggestions in reference to Aerial locomotion, portable engines, meteorological phenomena, clouds, landscapes, balloons, kites &c., &c. The local Secretary and corresponding agent for the United States is James W. Ward, Esqr., No 1 west 47th St, New York.

Missing numbers will be supplied to subscribers, without charge by giving notice, always giving their name distinctly with the full post-office address. Clergymen, Theological Students, Young Men's Christian Associations and Public Libraries, will be supplied with Hall's Journal of Health, for 1868, for sixty cents, if application is made before the first of August next.

HARPER'S MONTHLY.
HARPER'S WEEKLY.
HARPER'S BAZAAR.
ATLANTIC MONTHLY.
GALAXY.

Either of the above with Hall's Journal of Health will be furnished for one year for four dollars, the subscription to our Journal commencing with Jauuary ; last that of the others, from the time designated.

We guarantee the truthfulness of the advertisement of "Baldwin the Clothier" we know him of old.

THE LAW OF HUMAN INCREASE,
Or population based on physiology and psychology; by Nathan Allen, A. M. M. D. Lowell, Mass., 58pp, 8mo, sent post paid, for 40 cts. The Lowell daily Courrier says, " This is one of the most interesting and instructive essays." It is republished in the " Quarterly Journal of Psycholocical Medicine " for April, 1868. The Springfield Republican and Boston Commonwealth, elaborately review the pamphlet. The Republican says, " The work places Dr. Allen in the foremost rank of American writers, on the subject which he has chosen." The essay strives to account for the fact that the poor have so many more children than those who are in a better condition of life ; the question is well met and ably answered ; and merits wide attention.

" VULGARISMS," and other errors of speech, including a chapter on taste and one containing examples of bad taste, published by Claxton, Remson and Hafflefinger, 821 Market St, Philadelphia,Pa., a comely 12mo, of 194 pages, which ought to be in the library of every one, who desires to avoid those incorrect phrases and pronunciations which count so much against the culture, scholarship, and accomplishments of those who use them.

Our up town neighbors who want the monthlys, daily papers, and stationary generally would do well to patronize one of our very worthy citizens, P. Cummins, 805 Sixth Avenue, near 45th Street.

ARE YOU POSTED ? asks a brother editor in surprise at our MAY strictures on a popular monthly and some of its contributors ; and inquires further if we knew that one was orthodox ; another, an eminent minister's daughter, and a third, the son of a clergyman. Yes, we knew all this, and in addition that the Old Boy is the most orthodox individual of our acquaintance, in fact knows too much to be anything else than orthodox. It is an honor and a privilege to be the child of a clergyman and to be brought up under the influences of a minister's family, but sometimes, although not as often by any means as is generally supposed, ministers' children disgrace their paternity. The spirit of the times requires that we should take men and women as they are themselves and not for what their fathers have been. We honor and praise the names mentioned for their splendid talents and for whatever they have done and written and said in the interests of humanity and for the elevation of the race, but for their war against the sanctity of the marriage relation, for their making a butt, a bye-word and a jest of the ministry and for their stilettoing of that religion which makes, sustains and saves a nation, we praise them not; they are accountable to the bar of public opinion, the respect of all good men and the sterner realities of a dying hour and a judgment day, when recreancy to a man's religious faith and the buffooneries of his pen against religion, shall reap the remorses of a Julian and the judgments of an Iscariot. Whatever of talents and influence any man possesses, let him throw it on the side of religion, of the Bible and of the ministry of reconciliation, and then, at that great day, for which all other days were made, it shall be said by the Judge Omnipotent "ye did it unto me," "come up higher."

Brooklyn Boarding, at 32 Lafayette Avenue, Brooklyn, will be found all the comforts of a friendly home, under the management of one of the best house-keepers in the land, a lady of energy, who knows how to keep a tidy mansion, and a well spread table.

Miracles of Modern Surgery. As often as we call at Dr. Sayres' office, we are so impressed with the wonderful transformations which scientific surgery accomplishes for the human frame in effacing its deformities and correcting its unsightly contortions, club-feet, twisted spine, bent limbs, tumors, ghastly wounds and the like, that we are constrained to ask, "whereunto will these things grow"? and this note is made to invite any of our medical subscribers from the country, if they visit the city, to call on this eminent surgeon at his splendid rooms 285 Fifth Avenue, and they will find in this enthusiastic operator, the whole hearted welcome and courtesy of a gentleman; in truth we don't know which most to admire the genius of the operator in the nephew, or the christian and large-hearted liberalities of his uncle, the Kentucky Banker, David A. Sayre, the sterling Presbyterian and Patriot, at a time when patriotism was solid gold, among the hunters of Kentucky.

